# Distribution of Health Resource Allocation in the Fars Province Using the Scalogram Analysis Technique in 2011

**Published:** 2015-07

**Authors:** Nahid Hatam, Shahnaz Kafashi, Zahra Kavosi

**Affiliations:** School of Management and Information Sciences, Shiraz University of Medical Sciences, Shiraz, Iran

**Keywords:** Health status indicators, Iran, Health resource, Distribution

## Abstract

The importance of health indicators in the recent years has created challenges in resource allocation. Balanced and fair distribution of health resources is one of the main principles in achieving equity. The goal of this cross-sectional descriptive study, conducted in 2010, was to classify health structural indicators in the Fars province using the scalogram technique. Health structural indicators were selected and classified in three categories; namely institutional, human resources, and rural health. The data were obtained from the statistical yearbook of Iran and was analyzed according to the scalogram technique. The distribution map of the Fars province was drawn using ArcGIS (geographic information system). The results showed an interesting health structural indicator map across the province. Our findings revealed that the city of Mohr with 85 and Zarindasht with 36 had the highest and the lowest scores, respectively. This information is valuable to provincial health policymakers to plan appropriately based on factual data and minimize chaos in allocating health resources. Based on such data and reflecting on the local needs, one could develop equity based resource allocation policies and prevent inequality. It is concluded that, as top priority, the provincial policymakers should place dedicated deprivation programs for Farashband, Eghlid and Zaindasht regions.

## Introduction

One of the fundamental goals of any government is to provide public healthcare services. Health systems have four main functions, namely stewardship (policy and controlling), financing (collecting, pooling and income allocation), develop resources (human, knowledge, physical, and equipments), and provision and service delivery.^1^ Governments are responsible for providing health services for all; almost 5-10% of the GDP of most countries is allocated to the health sector budget.^2^

Healthcare resources and costs are probably the largest and the most uncontrollable budgetary issues. Consequently, policymakers must properly manage and control resources to maximize productivity.^3^ Despite the huge allocation of resources in this sector, yet, the conflict between scarce resources and unlimited needs is more prominent in the healthcare sector. Therefore, priority setting in the healthcare has emerged as an unpreventable task, though it is not restricted to the circumstances of scarce resources.

A prerequisite for optimal resource distribution is to recognize the existing conditions.^4^ The main step towards appropriate decision-making process by health policymakers is to rely on accurate data and to show flexibility when faced with new realities. A participatory approach in the priority setting of program evaluation may contribute to improved allocation and efficient use of scarce resources. This is particularly valid in low-income countries where such approach could assist in reflecting local needs based on resource allocation criteria in order to develop equity-based policies.^5^^,^^6^

Overall awareness about inequity in resource allocation distribution has an important role in healthcare sector.^7^ Tofighi et al. showed that inequity in geographic resource distribution resulted in accessibility to rural and remote areas.^8^ The result of a study in Uganda showed that inequity in resources (financial and human) created gaps in accessibility.^9^ The distance between health centers and remote areas reduce accessibility and ultimately cause inequity in that area. Moreover, reduced access of people in the remote areas results in deprived layers of society and unbalance in supply-demand of resources. This would lead to disharmony in distribution versus demand. Thus, improving healthcare access of people in such remote areas should be the main goal of policymakers.^10^^,^^11^

Researchers use quantitative techniques to design the distribution of resources. In case of inadequate information, allocating more time and financing of these techniques could be useful.^12^^,^^13^ One such technique is the scalogram in which an area is ranked based on Indicators. In this study, we used the analysis of the scalogram due to its simplicity, dynamics, flexibility to analyze structural indicators in the health sector, and the possibility to identify different zones.

It should be noted that the use of this technique in the healthcare system has been performed in few studies across Iran. The aim of this study was to investigate the use of health structural indices by the scalogram technique in the Fars province and to determine the developed and deprived areas. The result would offer a map that represents the development pattern and differences between the areas of Fars province towards a better planning, allocation of health resources and equity. 

## Materials and Methods

This study is a cross sectional study for system evaluation, which was approved by the Ethics Committee of Shiraz University of Medical Sciences. The following cities of Fars province were investigated in this study; namely Abadeh, Arsanjan, Estahban, Eghlid, Bvanat, Pasargard, Khorambid, Khonj, Darab, Zarindasht, Sepidan, Shiraz, Firozabad, Ghirokazin, Kazeroon, Larestan, Lamerd, Marvdasht, Mamassani, Mohr and Neiriz. Structural indicators, according to the WHO 2006 report,^14^ were categorized in three clusters: institutional indicators (n=8), human indicators (n=19), rural institutional indicators (n=3). The list of indicators is presented in table 1. Data were collected from the statistical yearbook of 2010 and entered in the SPSS software. After ranking and scoring, data were analyzed with the scalogram technique. The score of 1-4 was given to the classification of data (4: positive condition, 1: negative condition). In the case of positive condition, cities had effective statues. The cities were grouped into five development clusters and all scores were aggregated in which the values of 120 and 30 represented the maximum and minimum scores, respectively. We assessed distance gaps and amplitude of score changes (R) by the Sturgis formula:

$$\left(i=\frac{R}{\left(1+3.3logN\right)}\right)$$

i=Class interval

R=Range of classes

N=Number of items that should be classified category

Finally, GIS was used to display graphical and spatial map. 

## Results

The results related to the value of mixed indicators in the scalogram technique were ranked after the scoring was completed. The results are shown in three clusters.

*Institutional Indicator*

Cities were categorized and ranked into five regions:

1. Developed areas: Abadeh, Estahban, Khorambid, Ghirokazin, Larestan, Marvdasht and Mohr
2. Semi-developed areas: Arsanjan, Firozabad and Bavanat
3. Moderately developed areas: Eghlid, Darab, Shiraz
4. Less developed areas: Pasargard and Khonj
5. Underdeveloped areas: Zarindasht, Sepidan, Kazeroon, Lamerd, Mamassani and Neiriz

The indicator showed that 36.3% of the cities in the province were in good condition and 22.72% in bad condition. From this, the indicator related to health service centers had the highest score than other indicators, and the active treatment beds and drugstores per 1000 population had the lowest score in the institutional indicator.

*Human Resource Indicator*

The six cities in the developed area (Abadeh, Arsanjan, Estahban, Shiraz, Firozabad, Mohr, Neiriz, Pasargard, Ghirokazin and Lamerd) were to a certain extent developed and Khonj, Farashband, Mamasani and Marvdasht were moderately developed. The less developed areas were khorambied, Sepidan, and Darab. Larestan, Kazeroon, Bavanat, Eghlid and Zarindasht were ranked as underdeveloped. Among the indicators in table 1, the proportion of pediatricians per 1000 people had good condition in the indicator`s group and the proportions of pathologists per 10000 people were in bad condition.

**Table 1:** Structural indicators extracted from the statistical yearbook of Fars province

**Indicators (proportion per 1000 people)**	**Total**
Institutional	
Active treatment centers	56
Active beds at treatment centers	43
Active health centers	52
Clinical laboratory centers	49
Pharmacy	64
Radiography centers	34
Rehabilitation centers	61
Urban health centers	34
Human resource	
Internist	49
Cardiologist	40
Pediatricians	46
Psychiatrists	59
Dermatologist	58
General surgery specialist	40
Urologists	52
Orthopedist	49
Neurologist	46
ENT specialist	46
Eye specialist (ophthalmologist)	43
Gynecologist	52
Anesthesiologist	55
Radiotherapist	34
Pathologist	62
Dentist	43
Pharmacologist	52
Paramedical	37
Infectious diseases specialist	58
General practitioner	49
Rural Institutional	
Rural active health house	61
Rural active health center	31
Percentage of villages covered by rural active health house	55

*Rural Institutional Indicator*

The result of the indicator showed that, Bavanat, Darab, Sepidan, Mohr and Neiriz were the development areas. Arsanjan was to a certain extent a developed city, and Ghirokazin and Kazeroon were in the moderately developed category. In less developed group, there were seven cities; namely Eghlid, Pasargard, Khonj, Farashband, Firozabad, Marvdasht and Neiriz. Shiraz, Zarindasht, Khorambid and Estahban were in the underdeveloped group. Overall, according to the institutional indicators, 27.22% of the provincial cities were in a very good condition, 72.22% cities were in bad condition. The proportion of the rural active health houses per 1000 people had bad condition. In addition, the proportion of the rural active health centers per 1000 people had bad condition.

*Development of the Cities in the Fars Province*

The result of indicators in sample ranking with scalogram in provincial cities showed that, Mohr and Zarindasht had the highest score (85) and the lowest score (36), respectively. The statues in the Fars province by assessment scores showed that 14% of the cities were in less developed areas and 36% were in level of structural indicators. In this study, Mohr, Estahban, Abadeh, Ghirokazin, Arsanjan, Lamerd and Neiriz cities were among the developed areas (36% of the cities). It was observed that, four cities including Shiraz, Larestan, Firozabad and Marvdasht were to some extent developed (18% of the cities). Kazeroon, Bvanat, Pasargard, Khorambid, Sepidan, Khonj, Darab and Mamassani were in the moderately developed category (36% of the cities). In this study, two less developed cities were Eghlid, Farashband (9% of the cities). Zarindasht with 36 score was in the underdeveloped group (4% of the cities). 

Generally, in this study, the proportion of active beds at treatment centers and the proportion of drugstores per 1000 people were in the lowest statues, but the proportion of psychiatrists and rural active health houses per 1000 people were at the highest level.

**Figure 1:**
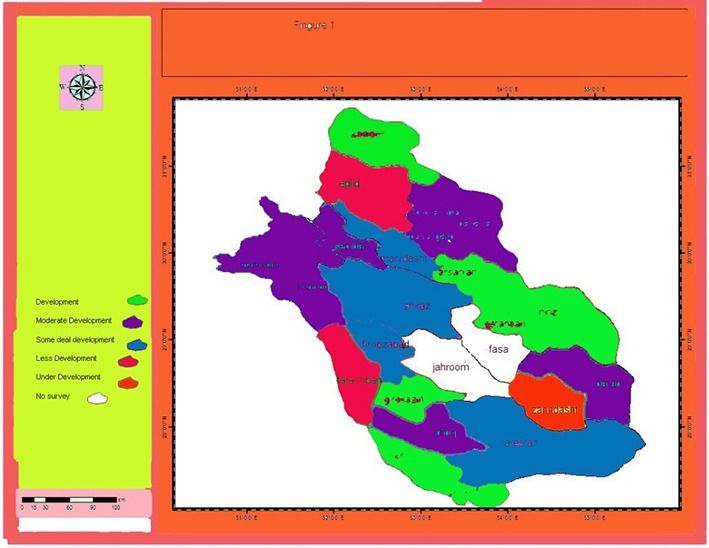
Status of development in the cities of Fars province. Note: The cities shown in white color (Fasa and Jahroom) are independent from Shiraz University of Medical Science and thus not included in this research.

Result ranking of Fars province for structural indicators (institutional, human resources and rural institutional) is shown in table 2. The GIS software was used to draw development status of healthcare indicators, as shown in figure 1.

**Table 2:** Status of development in the cities of Fars province according to health structural indicators

**No.**	**Cities**	**Percent**	**Classes distance**
1	Mohr, Estahban, Abadeh, Ghirokazin, Arsanjan, Lamerd and Neiriz	31.8	Development (89.4-75)
2	Shiraz, Larestan, Firozabad and Marvdasht	18.18	Semi development (64.5-74.9)
3	Kazeroon, Bvanat, Pasarghard, Khorambid, Sepidan, Khonj, Darab and Mamassani	36.8	Moderate development (64.4-54)
4	Eghlid, Farashband	9.09	Less development (53.9-43.5)
5	Zarindasht	4.45	Under development (43.5-33)
Total		100	

## Discussion

The main step in developing health sector and to reduce the gap between different areas, is to create awareness of regional needs and resource distribution. Evidence showed that, some areas are more developed than others are. If the planners can detect the important development factors, then the managers would optimally allocate resources to regions.^4^ According to the institutional indicators, the result showed that 50% of the Fars province was in the intermediate to underdeveloped level. Such statistics, in terms of the number of cities and the degree of development, highlight the need for a reallocation and revision of the health resources in this region.

Based on the results, the proportion of the health centers per 1000 people had a high score, but the active beds and pharmacies per 1000 people had a low score. Accessibility to a pharmacy is a critical index. Inadequate drug supplies and the number of pharmacies have a negative impact on people`s health. The proportion of pharmacies per 1000 people was in the lower level across all cities in the province. This issue is not only specific to Iran since even a US based study by Kaakeh et al.^15^ showed that drug scarcity had a negative impact on patients’ treatment. It is also shown that uneven distribution in geographic areas created problem for patients.^16^ It seems that, severe financial deficiency and rapid currency fluctuation have strengthened the role of the black market. Decentralization of managing such resources combined with giving incentive to pharmacists to work in the private sector could resolve this imbalance.

In this study, active treatment bed indicator in cities was in the lower level and the distribution was not balanced. Geographic techniques for such resource allocation and distribution can maximize accessibility in the region.^17^ To determine the number of required beds, criteria such as geography population, length of stay, occupied bed ratio should be used in order to create a balance between the demand and supply.^15^

The result of human indicators ranking showed that, 54.2% of cities are classified in the intermediate and underdeveloped level. Among these indicators, the proportion of paramedical per 1000 population was in ideal status. Similar to another study,^16^ the proportion of pathologists per 1000 people index was set in the low region in this study. Since human resource is a critical factor for service provider organizations, its planning could enhance health indicators. Inequity in the distribution of physicians in Iran, Japan, and America as well as the developing countries create accessibility problem for the local peoples. To confront the scarcity of pathologists and physicians in the Fars province, considering the importance of this index and its effect on development, it seems that equipping laboratories with advanced tools and offering incentive to pathologists and physicians to relocate to such areas would somehow resolve this problem.

Health resources in rural regions play an important and critical role. Lack of attention to local needs and the required human resources, create problems in developing rural regions. Inadequate public and private health services, lack of transportation network and communications, agricultural based industry, and lack of investment are the few barriers that demotivate the allocation of health resources. Current results showed that, 49.8% of cities in the province are in intermediate to underdeveloped level due to this index. Comparing with the previous indicators, this index had the highest score among the structural indicators, in other words, planning in this part lead to the improvement of this index. Poor services in this sector and uneasy access of the population to suppliers can be the main reason for deprivation of this region. Therefore, constructing health centers, rural active health houses, and health posts in these cities could solve the problem. Reallocation and relying on local cooperation would create a positive spin in reducing development gaps.

According to total indicators, the total development status of the Fars province showed 31.8% of cities in the province are developed, 18% developed to some extent, 36.8% intermediate, 9.09% less developed and 4.45% in the underdeveloped categories. 

## Conclusion

This study showed that there are gaps in the health structural indicators in the Fars province. This investigation offers an appropriate method to identify the development level of the province in terms of health structural indicators. With respect to the results, it emphasizes on the inequality of the development level among the health sectors across the region. The provincial and state policymakers must pay attention to these issues in allocating health facilities. Their plans should be configured towards reducing the gap in accessibility to healthcare facilities.

This study is based on the information obtained from the structural indicators; therefore, we recommend further studies to check the status of the distribution of other indicators at the provincial level to compare with the results from different techniques in the distribution of resources. 
